# A mild phenotype associated with *KCNQ1* p.V205M mediated long QT syndrome in First Nations children of Northern British Columbia: effect of additional variants and considerations for management

**DOI:** 10.3389/fped.2024.1394105

**Published:** 2024-05-31

**Authors:** Simona Bene Watts, Barbara Gauthier, Anders C. Erickson, Julie Morrison, Mavis Sebastian, Lawrence Gillman, Sarah McIntosh, Connie Ens, Elizabeth Sherwin, Rod McCormick, Shubhayan Sanatani, Laura Arbour

**Affiliations:** ^1^ Island Medical Program, University of British Columbia, Victoria, BC, Canada; ^2^ Epidemiology and Surveillance Unit, Interior Health Authority, Kelowna, BC, Canada; ^3^ Health Protection Branch, Ministry of Health, Victoria, BC, Canada; ^4^ Gitxsan Community Member, Hazelton, BC, Canada; ^5^ Department of Medical Genetics, University of British Columbia, Vancouver, BC, Canada; ^6^ Department of Pediatrics, Division of Cardiology, British Columbia Children’s Hospital, Vancouver, BC, Canada; ^7^ Department of Pediatrics, Children’s National Hospital, Washington, DC, United States; ^8^ Department of Education and Social Work, Thompson Rivers University, Kamloops, BC, Canada; ^9^ Division of Medical Sciences, University of Victoria, Victoria, BC, Canada

**Keywords:** long QT syndrome (LQTS), First Nations, Indigenous, carnitine palmitoyltransferase 1A, long QT syndrome type 1, *KCNQ1*

## Abstract

**Introduction:**

Congenital Long QT Syndrome (LQTS) is common in a First Nations community in Northern British Columbia due to the founder variant *KCNQ1* p.V205M. Although well characterized molecularly and clinically in adults, no data have been previously reported on the pediatric population. The phenotype in adults has been shown to be modified by a splice site variant in *KCNQ1* (p.L353L). The *CPT1A* p.P479L metabolic variant, also common in Northern Indigenous populations, is associated with hypoglycemia and infant death. Since hypoglycemia can affect the corrected QT interval (QTc) and may confer risk for seizures (also associated with LQTS), we sought to determine the effect of all three variants on the LQTS phenotype in children within our First Nations cohort.

**Methods:**

As part of a larger study assessing those with LQTS and their relatives in a Northern BC First Nation, we assessed those entering the study from birth to age 18 years. We compared the corrected peak QTc and potential cardiac events (syncope/seizures) of 186 children from birth to 18 years, with and without the *KCNQ1* (p.V205M and p.L353L) and *CPT1A* variants, alone and in combination. Linear and logistic regression and student *t*-tests were applied as appropriate.

**Results:**

Only the *KCNQ1* p.V205M variant conferred a significant increase in peak QTc 23.8 ms (*p* < 0.001) above baseline, with females increased by 30.1 ms (*p* < 0.001) and males by 18.9 ms (*p* < 0.01). There was no evidence of interaction effects with the other two variants studied. Although the p.V205M variant was not significantly associated with syncope/seizures, the odds of having a seizure/syncope were significantly increased for those homozygous for *CPT1A* p.P479L compared to homozygous wild type (Odds Ratio [OR]3.0 [95% confidence interval (CI) 1.2–7.7]; *p* = 0.019).

**Conclusion:**

While the *KCNQ1* p.V205M variant prolongs the peak QTc, especially in females, the *CPT1A* p.P479L variant is more strongly associated with loss of consciousness events. These findings suggest that effect of the *KCNQ1* p.V205M variant is mild in this cohort, which may have implications for standard management. Our findings also suggest the *CPT1A* p.P479L variant is a risk factor for seizures and possibly syncope, which may mimic a long QT phenotype.

## 1 Introduction

Long QT syndrome (LQTS) predisposes those affected to arrhythmia, syncope, seizures, and sudden cardiac death. While generally thought to be rare, affecting ∼1 in 2,000 individuals (1), congenital LQTS affects approximately 1 in 125 individuals in the Gitxsan community (2). The Gitxsan First Nation territory (People of the River Mist) covers a landmass of 33,000 square kilometres within northwest British Columbia (BC), Canada. The Gitxsan community voiced concern about the high rate of LQTS in 2004, and from this a community-based participatory research project was initiated. The project identified the novel pathogenic variant *KCNQ1* p.V205M as the primary cause of LQTS (3). Pathogenic variants in *KCNQ1* contribute to LQTS type 1 (LQTS1), the most common cause of LQTS. The effect of the p.V205M variant has been well characterized molecularly and within this First Nation adult population (3–5); however, the effect within the pediatric population has not been reported. Understanding the pediatric manifestations of this condition in the Gitxsan is imperative when considering the life course of the condition and relevant management strategies.

In addition to p.V205M, other genetic variants that may influence the presentation of the LQTS phenotype, or may mimic the phenotype, are present within the Gitxsan population. These include *KCNQ1* p.L353L, a synonymous splice site variant and modifier of the LQTS phenotype (5), *ANK2* p.S646F, a variant associated with structural heart disease and an adult onset form of LQTS (6), and *CPT1A* p.P479L, a variant associated with infant death (7, 8) and hypoglycemia (9–11). Not only is the *CPT1A* variant commonly found among Northern Indigenous populations in Canada (12) and BC First Nations (7), but hypoglycemia may present with symptoms (e.g., seizures) that can be difficult to differentiate from LQTS.

The variants that cause the cardiac phenotype of LQTS1 (*KCNQ1*) are also expressed in pancreatic islet cells and may predispose individuals to hypoglycemia (13). Moreover, beta-blockers are the mainstay treatment for LQTS and may alter the adrenergic response to hypoglycemia (14), and hypoglycemia itself has been observed to prolong the QT interval (15) and trigger arrythmia (16, 17). Over the last decade, at least three First Nations children in Northern BC with LQTS1 have presented with symptomatic hypoglycemia and a reduced level of consciousness (Table 1). All children were either heterozygous or homozygous for *CPT1A* p.P479L and taking beta-blocker medication, leading to the concern that the combination of the p.V205M and common *CPT1A* variant when inherited together might present with a more severe or varied phenotype.

**Table 1:** Case presentation of three Northern BC First Nations children with symptoms of hypoglycemia and reduced levels of consciousness.

Case	Sex	*KCNQ1* variant	*CPT1A* variant	Age^a^	Clinical presentation	Illness (Y/N)^b^	Beta-blocker
1	M	p.V205M positive	p.P479L homozygote (PP)	4 years	Low level of consciousness: unable to rouse from sleep in morning, blood glucose 2.6 mmol/L; previous history multiple febrile seizures at ages 5 months and 4 years.	Y	Nadolol
2	F	p.V205M positive	p.P479L homozygote (PP)	4 years	hypoglycemic seizure: onset 5 am during sleep, blood glucose 1.6 mmol/L	N	Propranolol
3	F	p.V205M positive	p.P479L heterozygote (PL)	3.5 years	hypoglycemic seizure: onset 7 am when asking for breakfast, blood glucose 1.6 mmol/L	N	Nadolol

M, male; F, female.

^a^ Age at clinical presentation of hypoglycemic episode in years.

^b^ Intercurrent illness at the time of hypoglycemic episode.

Founder populations provide a unique opportunity to study the effect of genetic variants within a relatively similar population. We sought to understand the effect of the *KCNQ1* p.V205M and p.L353L variants within the pediatric population and whether events consistent with a LQTS phenotype were influenced by the presence of the *CPT1A* p.P479L variant. Because of the unclear cardiac effect of the *ANK2* p.S646F variant which is also present in this community, we opted to exclude those with the variant in this analysis.

## 2 Methods

### 2.1 Enrollment and data collection

As part of a larger community-based longitudinal research study (3), participants were invited to enroll if they had clinical features of LQTS or were related to an individual with LQTS. Community approval and participant/parent informed consent and assent was obtained. Upon enrollment, a health information questionnaire was administered, medical records reviewed, a comprehensive family history recorded, and a 12-lead electrocardiogram (ECG) performed. Events leading to loss of consciousness (LOC) including syncope or seizure were recorded. Participants from birth until age 18 years of age and enrolled from 2005 to 2019 were included. Ongoing outpatient cardiology visits to BC Children's Hospital or Northern outreach clinics were recorded on all p.V205M positive participants, and negative participants were referred for cardiology related symptoms.

#### 2.1.1 Exclusions

Participants 18 years and older at time of enrollment, without an ECG, missing genotyping data for any of the variants under study (*KCNQ1* p.V205M, *ANK2* p.S646F or *CPT1A* p.P479L), *ANK2* p.S646F positive, or with a potentially confounding medical diagnosis, were excluded. For the *KCNQ1* p.L353L specific analysis, those with unknown variant status were excluded (*N* = 2). For the QTc analysis, only those related to participants with the p.V205M variant were included as controls to minimize ascertainment bias (78 were excluded because they were not related to a case). Of note, two p.V205M positive females were excluded because their QTcs were outliers potentially skewing results, but the cases are described below.

### 2.2 Ethics

Research ethics approval was received by the University of British Columbia Clinical Research Ethics Board, Northern Health Authority Research Ethics Board, Island Health Research Ethics Board, and the University of Victoria Research Ethics Board. The Gitxsan Health Society Board of Directors approved the research and a local research review committee participated in the research design and reviewed results. The results were also presented publicly for Gitxsan community members.

### 2.3 Genotyping

Blood or saliva was collected for DNA sequencing under the stipulation of *DNA on Loan* (18). Genotyping for the *KCNQ1* variants p.V205M and p.L353L was performed at the Molecular Genetics Laboratory at the BC Children's & Women's Hospital via clinical grade Sanger sequencing. Research testing for *CPT1A* p.P479L and *ANK2* p.S646F variants was performed at the Centre for Applied Genomics at The Hospital for Sick Children in Toronto.

### 2.4 QTc determination

For those p.V205M positive and their 1st to 3rd degree relatives, all available ECGs from birth to their eighteenth birthday were included (ECG upon enrollment and any previous or subsequent ECGs taken during childhood). All ECGs were manually read and the results denoted as mQTc. The QT interval was measured in lead II or V5 using the tangent method (19) by at least one of two pediatric cardiologists (SS and ES) blinded to clinical and variant status. The QT interval corrected for heart rate (QTc) was then calculated using Bazett's formula (20). When the ECG was read by more than one practitioner, the average of the two final QTc measurements was calculated. If the difference between final QTc measurements was greater than 30 ms, the ECG was discussed and the final measurement agreed upon by consensus.

### 2.5 Statistical analysis

The highest mQTc measured during childhood was identified as the participant's “peak” mQTc. The mean peak mQTc between p.V205M positive cases and negative relatives (see below for description) was compared using descriptive analyses and *t*-tests. Univariate, bivariate, and multivariate linear regression (Ordinary Least Squares) was then used to explore the association of genetic variants, independently and in combination, on peak mQTc. Sex was also included as a variable.

Additionally, *t*-tests were performed to compare the highest mQTc recorded from p.V205M positive cases and negative controls within defined age categories (<1 year old, 1–4 years old, 5–9 years old, 10–14 years old, and 15–17 years old). Univariate and multivariate binomial logistic regression analysis were used to calculate the odds ratio [OR] between the genetic variants on LOC events considering the *KCNQ1* p.V205M and *CPT1A* p.P479L variants, independently and in combination. Logistic regression was also used to calculate the odds of LOC events by mQTc. *STATA 16-IC* and *GraphPad Prism* were used to perform the statistical analyses.

## 3 Results

### 3.1 Participants

A total of 276 pediatric participants were enrolled in the study. Eighty-seven were excluded because they had no ECGs for analysis or genotyping was incomplete. Three children were excluded from the analysis due to confounding medical circumstances. Two of the three children had the p.V205M variant and their QTcs were considered outliers. Of those, one had evidence of left ventricular non-compaction cardiomyopathy (LVNC) and the other had a mixed connective tissue disorder which was complicated by an overdose with a QT prolonging drug (hydroxychloroquine). Their QTc values ranged from 580ms-622 ms and were excluded from the QTc analysis because of the potential of skewing the results. The third exclusion was p.V205M negative and had Wolff-Parkinson-White syndrome.

For the QTc analysis, there were 40 p.V205M heterozygotes (referred to as “V205M positive”) and no p.V205M homozygotes. There were 68 p.V205M negative 1–3rd degree relatives. Of the 40 participants who were p.V205M positive, 38 were confirmed to be positive through family cascade genetic testing, of which three were shown to have a prolonged QTc on ECG prior to genetic testing. Two participants presented symptomatically and had subsequent genetic testing. All 40 participants were advised beta-blocker therapy, with 35/40 prescribed beta-blockers at diagnosis. Compliance of beta-blocker therapy was not assessed in this study. All had a family history of LQTS. For this analysis, a total of 214 ECGs were manually read (mQTc) on 108 participants.

For the LOC analysis, all p.V205M positive cases including the QTc outliers, 67 of their 1–3rd degree relatives, and 78 unrelated individuals who were enrolled in the study were included. Two individuals (one p.V205M positive and one p.V205M negative) were removed due to confounding neurological conditions. Additionally, two individuals reported “possible” seizures and were therefore excluded from the seizure-specific analysis. See Supplementary Figure S2 for details.

The mean age of those p.V205M positive was 7.6 years (SD = 5.8) at enrollment, and they were followed for a mean of 5.8 years (range of follow up 0–13.1 years) by cardiology services. The mean age at enrollment for p.V205M negative participants was 8.7 years (SD = 4.8). Cardiology follow-up was minimal as would be expected (8 months); however, 46 ECGs on p.V205M negative individuals were on record for a period of up to 9.6 years post enrollment. Participant demographics are further described in Tables 2, 3.

**Table 2:** Participant demographics of QTc analysis (*n* = 108).

Characteristic	*N* ^a^
KCNQ1 (p.V205M)	
+ (heterozygous)	40
−	68
Sex	
Male	59
Female	49
Age at enrollment	
<1	7
1–4	33
5–9	23
10–14	34
15–18	11
Peak mQTc	
<440	50
440–460	38
>460	20
Number of ECGs	
1	62
2–5	39
6–10	5
11	2
KCNQ1 (p.L353L)	
+ (heterozygous)	6
−	100
Unknown	2
CPT1A (p.P479L)	
PP	26
PL	53
LL	29
p.V205M *p.L353L	
+*+	2
+*−	38
−*+	4
−*−	62
p.V205M *p.P479L	
+*PP	10
−*PP	16
+*PL	17
−*PL	36
+*LL	13
−*LL	16
p.V205M by age category	
<1	
+	9
−	8
1–4	
+	18
−	21
5–9	
+	11
−	20
10–14	
+	12
−	24
15–17	
+	7
−	5

^a^ Non-relatives excluded from QTc analysis.

**Table 3:** Participant demographics of LOC analysis (*n* = 186).

Characteristic	*N* ^a^
History of LOC	
Syncope	32
Febrile seizure	13
Non-febrile seizure	15
All seizures	28
LOC (combined)^b^	50
Sex	
Male	94
Female	92
Age at enrollment	
<1	9
1–4	49
5–9	39
10–14	66
15–18	23
Relative	
Relatives to p.V205M	67
Non-relative to p.V205M	78
KCNQ1 (p.V205M)	
+ (heterozygous)	41
−	145
KCNQ1 (p.L353L)	
+ (heterozygous)	18
−	165
Unknown	3
CPT1A (p.P479L)	
PP	48
PL	92
LL	46
p.V205M *p.P479L	
+*PP	10
−*PP	38
+*PL	18
−*PL	74
+*LL	13
−*LL	33

^a^ 2 children excluded due to confounding neurological medical history and 1 child excluded because seizures reported as “possible” and did not experience syncope.

^b^ Loss of Consciousness (LOC) combined includes individuals with any type of seizure, syncope, or both seizure and syncope.

### 3.2 Peak QTc in childhood

**Figure 1:**
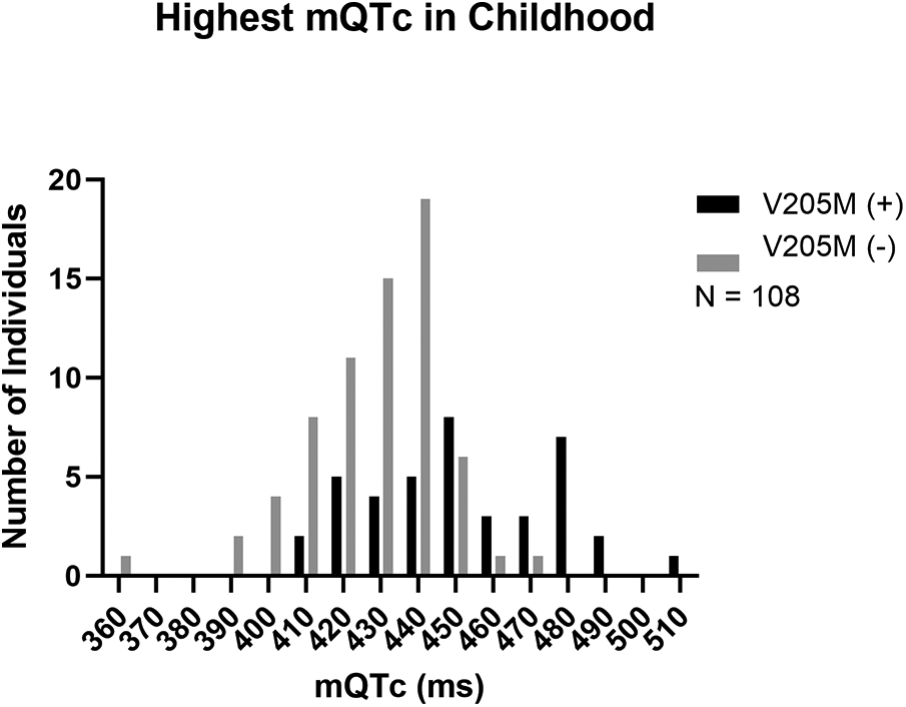
Peak mQTc in childhood. The peak mQTc measured in childhood from p.V205M positive children is depicted in black and the peak mQTc measured in childhood from negative control children is depicted in grey.

Figure 1 shows the distribution of those *KCNQ1* p.V205M positive participants compared to those without the p.V205M (mean QTc = 451.5 ms ± 24.3 ms and 427.1 ms ± 18.6 ms respectively *p* < 0.0001).

Regression analyses (Table 4) showed an average increase of QTc from baseline by 23.8 ms (95% CI: 15.3–32.5, *p* < 0.001) which was greater in females (30.1 ms) than males (18.9 ms), each significantly higher than baseline (*p* < 0.001), but not significantly different from each other. When added to the model, the p.L353L variant demonstrated a non-significant association of 15.5 ms among males and 9.1 ms among females. There was no association between QTc and the p.P479L variant (Supplementary Table S1).

**Table 4:** Linear regression analysis of variant association on peak QTc in childhood.

Variable	Beta coefficient^a^	95% CI	*p*-value
A) Males and Females, *n* = 106, intercept = 426.3 ms, adjusted *R*^2 ^= 0.22			
p.V205M	23.8	15.3–32.5	<0.001
p.L353L	9.1	−12.5–30.6	0.404
p.V205M *p.L353L	6.4	−31.0–43.8	0.735
Female sex	1.2	−7.1–9.3	0.792
B) Males, *n* = 59, intercept = 428.0 ms, adjusted *R*^2 ^= 0.20			
p.V205M	18.9	8.0–29.8	0.001
p.L353L	15.5	−13.1–44.2	0.281
p.V205M *p.L353L	3.4	−37.4–44.2	0.868
C) Females, *n* = 47, intercept = 425.5 ms, adjusted *R*^2 ^= 0.30			
p.V205M	30.1	16.0–44.1	<0.001
p.L353L	3.0	−30.4–36.5	0.856
p.V205M *p.L353L	–	–	–

^a^ Beta coefficients from OLS linear regression representing the baseline (intercept) and change in QTc (ms).

### 3.3 Intra-patient variability

**Figure 2:**
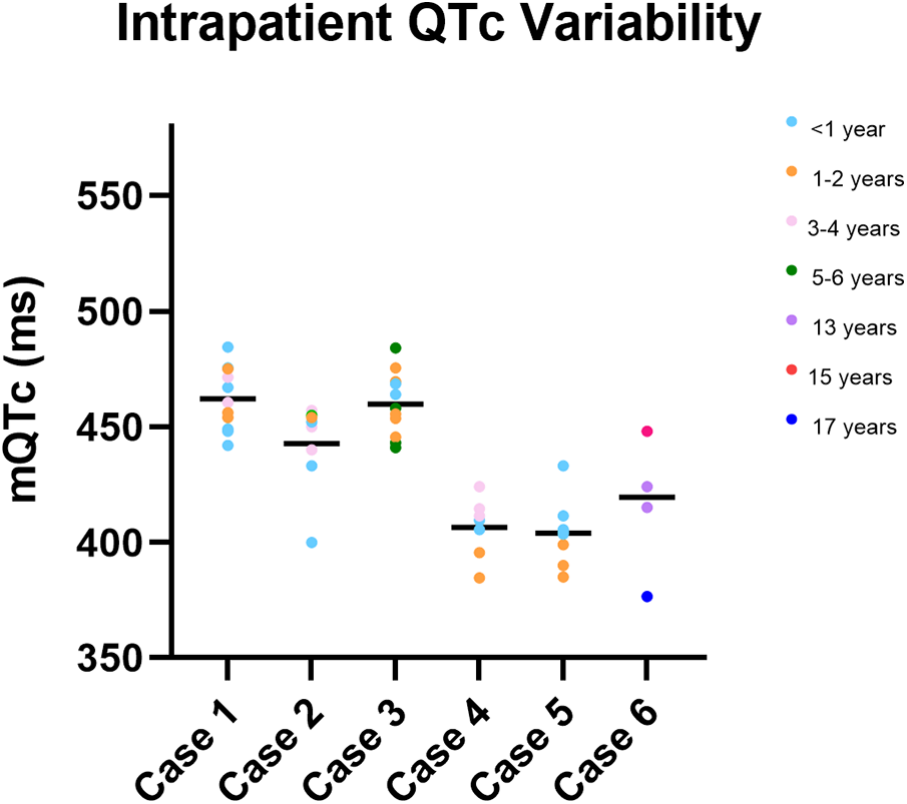
Intra-patient QTc variability in childhood. Depiction of manual QTc measurements recorded in childhood for six children (cases 1–6). Manual QTc measurements are depicted by coloured circles, with the mean mQTc measurement depicted by a black bar. The colour of the circle indicates the age at which the QTc was measured. Significant variability in mQTc measurements throughout childhood are observed in each child.

Figure 2 presents manual QTc measurements on repeat ECGs recorded in childhood at different ages for six cases. These cases were chosen as examples to demonstrate intra-patient variability in QTc throughout childhood.

### 3.4 Highest QTc by age category

Results comparing the highest QTc obtained from participants within five age categories (males and females combined) are shown in Supplementary Figure S1. In all age categories, the mean QTc was prolonged for those who were p.V205M positive, but statistically significant in the 1–4 year, 5–9 year, and 10–14 year age categories. Although the QTc was generally more prolonged in the remaining two categories, the differences were not statistically significant with smaller sample sizes.

### 3.5 Syncope and seizures

No statistically significant association between p.V205M and the odds of experiencing at least one LOC event in childhood was observed (OR = 1.3, 95%CI 0.6–2.9) (Table 5A1). However, the odds were 3.0 times higher in children who were homozygous for the *CPT1A* variant compared to children who were negative (95% CI 1.2–7.7) (Table 5A2). Of note, the odds of experiencing a seizure were >4 for those homozygous for the p.P479L variant compared to those negative (95% CI 1.1–17.2) and 3.0 times higher for those heterozygous for p.P479L with a trend towards statistical significance (95% CI 0.8–10.8) (Table 5C2). When stratified by seizure type, the odds of experiencing a non-febrile seizure were 7.4 (95% CI 0.9–64.3) and febrile seizure were 2.3 (95% CI 0.4–13.2) for those homozygous for the p.P479L variant compared to negative cases (Supplementary Table S2). There was no evidence of interaction between the two variants.

**Table 5:** Logistic regression analysis of LOC events.

Model	OR	95% CI	*p*-value
A) LOC^a^			
A.1) V205M model, *N* = 185			
V205M	1.3	0.6–2.9	0.445
A.2) CPT1A model, *N* = 185			
PL	1.4	0.6–3.3	0.466
LL	3.0	1.2–7.7	0.019
A.3) Integrative model, *N* = 185			
V205M	1.3	0.6–2.7	0.571
PL	1.4	0.6–3.3	0.462
LL	3.0	1.2–7.6	0.021
B) Syncope			
B.1) V205M model, *N* = 186			
V205M	0.8	0.3–2.1	0.622
B.2) CPT1A model, *N* = 186			
PL	1.2	0.4–3.2	0.789
LL	2.8	0.9–8.0	0.063
B.3) Integrative model, *N* = 186			
V205M	0.7	0.3–1.9	0.491
PL	1.1	0.4–3.2	0.795
LL	2.8	1.0–8.3	0.057
C) All seizures^b^			
C.1) V205M model, *N* = 183			
V205M	1.5	0.6–3.7	0.397
C.2) CPT1A model, *N* = 183			
PL	3.0	0.8–10.8	0.100
LL	4.4	1.1–17.2	0.033
C.3) Integrative model, *N* = 183			
V205M	1.4	0.5–3.5	0.472
PL	3.0	0.8–10.9	0.098
LL	4.3	1.1–16.9	0.037

PP, Homozygous wildtype for *CPT1A*, baseline *CPT1A* measurement in regression model; PL, Heterozygous for *CPT1A* p.P479L; LL, Homozygous for *CPT1A* p.P479L.

^a^ One participant removed that reported LOC event (seizure) as “possible”.

^b^ Three participants removed that reported seizures as “possible”.

Table 6 presents the number of children who experienced at least one LOC event in childhood stratified by p.V205M and p.P479L genotype. Of those without either variant (p.V205M negative and p.P479L negative), 10% experienced syncope and 3% seizures, which is similar to the general population rates (21, 22).

**Table 6:** Number of participants that experienced at least one LOC event in childhood by genotype.

Genotype		LOC (*n* = 185)^a^			Seizures (*n* = 183)^b^			Febrile S. (*n* = 183)^b^			Non-Febrile S. (*n* = 183)^b^			Syncope (*n* = 186)		
		No	Yes	%	No	Yes	%	No	Yes	%	No	Yes	%	No	Yes	%
V205M	(−)	107	37	26	122	20	14	133	9	6	131	11	8	119	26	18
	(+)	28	13	32	33	8	20	37	4	10	37	4	10	35	6	15
CPT1A	PP	39	9	19	45	3	6	46	2	4	47	1	2	42	6	13
	PL	69	22	24	76	15	16	84	7	8	83	8	9	79	13	14
	LL	27	19	41	34	10	23	40	4	9	38	6	14	33	13	28
PP*V205M	(−)	33	5	13	37	1	3	38	0	0	37	1	3	34	4	11
	(+)	6	4	40	8	2	20	8	2	20	10	0	0	8	2	20
PL*V205M	(−)	55	18	25	60	13	18	66	7	10	67	6	8	64	10	14
	(+)	14	4	22	16	2	11	18	0	0	16	2	11	15	3	17
LL*V205M	(−)	19	14	42	25	6	19	29	2	6	27	4	13	21	12	36
	(+)	8	5	38	9	4	31	11	2	15	11	2	15	12	1	8

PP, Homozygous wildtype for *CPT1A*; PL, Heterozygous for *CPT1A* p.P479L; LL, Homozygous for *CPT1A* p.P479L.

^a^ One participant removed that reported LOC event (seizure) as “possible”.

^b^ Three participants removed that reported seizures as “possible”.

Supplementary Table S3 depicts odds of LOC events by mQTc length among those p.V205M positive. Although there was an increased OR compared to reference (440 ms or less), there was no statistically significant difference for the categories with an increased mQTc length.

## 4 Discussion

### 4.1 Peak QTc

Although our results support that p.V205M significantly prolongs the QTc above baseline, with females notably more prolonged than males, our results suggests that those identified through cascade genetic testing (the majority of our cohort) have a mild phenotype. Only four cases presented prior to family screening. Two of these p.V205M positive cases were extreme outliers for the QTc analysis and thus excluded, including one who suffered a torsades de pointes/ventricular fibrillation cardiac arrest with an overdose of hydroxychloroquine. The second excluded case exhibited a high load of premature ventricular contractions with non-sustained ventricular tachycardia and was noted to have echocardiogram findings consistent with left ventricular non-compaction of unclear etiology (congenital vs. acquired left ventricular remodeling). Broad cardiac panel genetic testing (>130 genes) did not reveal any additional genetic contributors suggesting another diagnosis. For the remaining children, the majority were identified through cascade genetic testing (95%), and the mean QTc of 451.5 ± 24.26 ms is considered within the “borderline” category (440–460 ms) of pediatric QTc intervals (23). Our results suggest that the p.V205M variant has less of an effect in the pediatric age group than reported in our adult cohort. The mean QTc for variant positive adults was 476 ± 35 ms (4), suggesting a “prolonged” QTc (>470 ms) for adults (23). Similar to Gitxsan adults, the p.V205M variant in children was found to have a more clinically relevant effect on females compared to males, and that effect becomes more apparent in the 10–14 year old age category (see Supplementary Figure S1). Our data suggests that the effect of the p.V205M is generally mild in childhood and is corroborated by little evidence of historic pediatric deaths through our pedigree analysis.

A milder phenotype throughout childhood is consistent with the multigenerational presence of the variant. In circumstances with a severe presentation in childhood, reduced survival might impact population frequency of the variant. This phenomenon of a milder phenotype in founder populations in general with LQTS has been previously reported. Similar findings have been shown in Finland, where children with *KCNQ1* and *KCNH2* founder variants have been associated with significantly fewer cardiac events than children with Finnish non-founder *KCNQ1* and *KCNH2* variants (24). It is well known that proband presentations are more severe than those detected with cascade genetic testing and this effect can be exhibited in founder populations. Our founder population shows similar characteristics to the Finnish reports.

Although we did not find statistical evidence that the *KCNQ1* p.L353L variant prolongs the QTc in childhood, in contrast to our previous studies of adults, the sample size of children with p.L353L was small (*N* = 6) and likely underpowered to show any effect. Similarly, no effect of p.P479L alone or interaction effect between p.V205M and p.P479L on the QTc was observed. If there is an effect on the QTc due to hypoglycemia, it might be intermittent, and therefore not detected in our results because of the timing of ECGs.

### 4.2 Syncope, seizure and cardiac arrest

There was no significant increase in odds of syncope or seizures, alone or in combination with the p.V205M variant. We observed the rate of syncope to be low (15%) in those who are positive, which is consistent with most previous literature on the topic (0%–9.3%) (24–26). One study of note demonstrated a rate of 27%, which was observed in a study cohort exclusively composed of children with LQTS1 not taking beta-blockers (27). The majority of studies reporting the rate of cardiac arrest in pediatric LQTS1 patients have found it to be low, ranging from 0 to 1.6% (24–30). There was one exception suggesting an 8% risk of cardiac arrest, although there were only 12 LQTS1 individuals in this study. In that study, high risk infants with LQTS identified with torsades de pointes or heart block as study inclusion criteria were assessed (31). Within our cohort, as mentioned above, one p.V205M positive case with an overdose of a QT prolonging drug experienced torsades de pointes and a ventricular fibrillation arrest (1/42 or 2.4%). Notably, there were no cardiac events with typical daily activities, sleep, or exercise. However, most of our cases were identified through cascade testing and although most were prescribed beta-blockers, adherence was not documented.

Overall report of seizures was common within our cohort, ranging from 14% in the p.V205M negative participants to 20% in those positive (Table 6). Our results showed an association of the *CPT1A* p.P479L variant with seizures and possibly syncope. Although the variant's impact on fatty acid oxidation predisposing children to hypoglycemia is the most likely explanation, the role of *CPT1A* in the brain has not been well explored; therefore, other mechanisms cannot be excluded at this time. Previous studies have found an association between p.P479L and hypoglycemia (9–11), and LOC events (most commonly seizures) are a known manifestation of hypoglycemia. To our knowledge, there is only one previous report (a published abstract) of an association between seizures and the p.P479L variant. This study found that p.P479L homozygotes were more likely to experience non-febrile seizures compared to heterozygote and wildtype children (32). Our study corroborates that work, but more research is indicated. We are not aware of previous studies reporting an association between syncopal events and p.P479L. While hypoglycemia is known to be a rare cause of syncope (33), it is also possible the association is due to participants misreporting of the events documented with the medical history questionnaire. Further study and more precise delineation of syncope or seizure phenotypes is indicated in all age groups. Several children were enrolled into the study with suspicion of LQTS because they presented with syncope/seizures. Our evidence suggests that in Northern BC, the *CPT1A* p.P479L variant is more likely to contribute to syncope and seizures than the p.V205M variant. Given the *CPT1A* variant is common in BC First Nations and other Northern/Arctic populations, these results may be useful for other northern populations.

### 4.3 Pediatric LQTS management

Beta-blocker induced hypoglycemia has been observed in LQTS children (14) and hypoglycemic syncope may occur as a rare side-effect which risks being misinterpreted as an arrhythmic event (33). Those with the *CPT1A* p.P479L variant may be at increased risk for hypoglycemia, especially during illness or prolonged fasting, and beta blocker medication could increase the risk for a hypoglycemic event. Given that those with loss of function variants in *KCNQ1* may be prone to post-prandial hypoglycemia due to increased insulin release from the pancreas (13), increased vigilance is warranted when prescribing beta-blockers to those with both *KCNQ1* variants and the *CPT1A* p.P479L variant. In this population, clinicians counsel families about avoiding prolonged periods of fasting and ensuring adequate glucose intake during intercurrent illness.

Given that cardiac arrest is rare in the pediatric population and there is no evidence to suggest the p.V205M variant confers increased risk for seizures/syncope, we suggest that the clinical management of children with p.V205M may not always require beta-blockers. As noted, two of our more severe cases were detected symptomatically; however, those detected through cascade genetic testing showed only mildly increased QTcs. Given that most cases were diagnosed at young ages because of a family member presenting, provided with advice to avoid QT prolonging medications, and prescribed beta-blocker therapy, the management may have mitigated more severe childhood presentations. It is also possible that this population will have a less severe presentation given the milder presentations often documented with founder variants traced back for several generations. In accordance with well-established management standards most children with LQTS1 will be prescribed beta-blocker medications (34, 35). The AHA/ACC/HRS guidelines recommend beta-blockers to all LQTS patients with a QTc ≥470 ms and state that it is reasonable for asymptomatic molecularly diagnosed individuals with a QTc <470 ms to take beta-blockers (class IIa recommendation). The guidelines state that the benefits of beta-blockers are greater than the risks for asymptomatic individuals with a QTc <470 ms (35). However, it has also been suggested that beta-blockers may be unnecessary for some asymptomatic children (24, 36). Waddell-Smith et al. (2015) proposed that beta-blockers may not be essential if: “(1) the QTc is less than 470 ms and, (2) the patient does not have a C-loop LQTS type 1 missense mutation and, (3) the patient does not partake in high risk activities, and (4) the patient is either a preschool boy or prepubertal girl”. Additionally, due to the mild phenotype observed in children with Finnish founder variants, Finnish pediatric cardiologists suggest children with *KCNH2* founder variants start beta-blockers later in childhood but before the onset of puberty, provided their QTc is below 470 ms and there is no family history of cardiac arrest (24). Intentional non-therapy has also been proposed for asymptomatic adults with an older age at diagnosis and a QTc <470 ms (37).

In many circumstances, the evidence supporting beta-blocker therapy as a life-saving medication is firmly established (38); however, in circumstances that are less clear, shared-decision making with parents and older children could be considered. Although our sample size is relatively small, we posit that beta-blockers could be optional for p.V205M positive patients if they are asymptomatic, consistently measure a QTc <470 ms (note intra-patient variability of QTc is typical, see Figure 2), do not participate in high-risk activities, do not require treatment with potentially QT prolonging medications, and have no close family history of cardiac arrest. These decisions would of course follow the principles of shared decision making, in which the patient and care providers are fully informed about treatment pathways, risks, and benefits of each option. We also emphasize the importance of a safety plan, which includes supervised swimming, communal CPR awareness, and automated external defibrillator access. It is important to note that in our umbrella study documenting the family histories of more than 150 p.V205M positive cases of all ages, women of childbearing years have been observed to be at highest risk for cardiac arrest. However, only one cardiac arrest before age 18 has been reported to date.

In general, for those identified by family cascade testing with the p.V205M variant, we suggest avoidance of QT prolonging drugs and yearly (or more often as indicated) ECG QTc review (34, 35) by an informed pediatrician or cardiologist. The merits of beta-blocker medications should be discussed for all and recommended for those with a documented QTc greater than 470 ms, identified symptomatically, and participating in competitive sports. Self-awareness of cardiac symptoms (palpitations, syncope) and seeking medical attention as needed will reduce risk of events (34, 35). In addition, replenishing electrolytes in periods of dehydration (such as gastroenteritis and vigorous sports activities) is important to consider.

Although none of the children in this study were homozygous for the p.V205M variant, previous documentation of the clinical phenotype of homozygous cases suggest a more severe phenotype which includes pediatric onset of clinical features (4). As such, we recommend beta-blockers for any children homozygous for the p.V205M variant as well as close clinical monitoring, given additional management (such as implantable cardioverter defibrillator) may be necessary.

Although the *CPT1A* p.P479L variant is specific to Indigenous populations, our results are a reminder that genetic variants other than those contributing to LQTS may influence the phenotype. Given the high rate of the *CPT1A* p.P479L in British Columbia First Nations and other Northern Indigenous populations, more study should be carried out to better understand the apparent associated risk of syncope and seizures and consider potential preventative strategies.

## 5 Conclusion

The *KCNQ1* p.V205M variant was found to significantly prolong the QTc in childhood in the Gitxsan population; however, there was no statistical evidence that the p.V205M variant increased the odds of syncope/seizures or other cardiac events in childhood. In contrast, the *CPT1A* p.P479L variant, which is also common in this population, was found to significantly increase the odds of LOC events in childhood which may be misinterpreted as cardiac events. More research is needed to further elucidate the individual and combined effects of these variants. That most cases with the p.V205M variant are asymptomatic during childhood is reassuring. Although QT prolonging drugs should be avoided for all children with the p.V205M variant, other management strategies for those who are p.V205M positive, such as beta-blocker therapy, could be considered on a case-by-case basis.

## Data Availability

The original contributions presented in the study are included in the article/**Supplementary Material**, further inquiries can be directed to the corresponding author.
