# Supplementary material for: A mild phenotype associated with KCNQ1 p.V205M mediated long QT syndrome in First Nations children of Northern British Columbia: effect of additional variants and considerations for management
Source: Front Pediatr. 2024 May 31;12:1394105. doi: 10.3389/fped.2024.1394105 (PMC11176454; doi:10.3389/fped.2024.1394105)
Supplement: Supplementary file 1 [file Table1.docx]

**Supplementary Table 1.** Linear regression analysis of the *CPT1A* p.P479L effect on QTc in childhood (0-18yrs).

| **Variant** | **Beta Coefficient** | **95% CI** | **p Value** |
| --- | --- | --- | --- |
| A) p.P479L, n=108, intercept=439.4ms, adjusted R^2^=-0.01 | | | |
| PL | -6.0 | -17.4 to 5.4 | 0.299 |
| LL | 0.02 | -12.9 to 13.0 | 0.997 |
| Sex | -0.8 | -10.0 to 8.4 | 0.864 |
| B) p.P479L and p.V205M, n=108, intercept=429.7ms, R^2^=0.22 | | | |
| p.V205M | 24.0 | 15.7 to 32.4 | <0.001 |
| PL | -4.5 | -14.5 to 5.5 | 0.375 |
| LL | -1.6 | -12.9 to 9.7 | 0.783 |
| Sex | 0.3 | -7.8 to 8.4 | 0.944 |
| C) p.P479L*p.V205M, n=108, intercept=434.0ms, R^2^=0.23 | | | |
| p.V205M | 12.2 | -4.5 to 29.0 | 0.151 |
| PL | -11.2 | -23.7 to 1.3 | 0.078 |
| LL | -5.9 | -20.6 to 8.8 | 0.427 |
| p.V205M*PL | 18.6 | -2.3 to 39.4 | 0.080 |
| p.V205M*LL | 11.3 | -11.6 to 34.1 | 0.331 |
| Sex | 1.0 | -7.0 to 9.1 | 0.798 |
| PP – homozygous wildtype for *CPT1A* p.P479L, baseline *CPT1A* measurement in regression model  PL – heterozygous for *CPT1A* p.P479L  LL – homozygous for *CPT1A* p.P479L | | | |
