# Supplementary material for: A mild phenotype associated with KCNQ1 p.V205M mediated long QT syndrome in First Nations children of Northern British Columbia: effect of additional variants and considerations for management
Source: Front Pediatr. 2024 May 31;12:1394105. doi: 10.3389/fped.2024.1394105 (PMC11176454; doi:10.3389/fped.2024.1394105)
Supplement: Supplementary file 2 [file Table2.docx]

**Supplementary Table 2.** Logistic regression analysis of febrile and non-febrile seizures.

| **Logistic Regression Analysis of LOC Events** | | | |
| --- | --- | --- | --- |
| **Model** | **OR** | **95% CI** | **p Value** |
| **A) Febrile Seizures**† |  |  |  |
| D.1) V205M model, N=183 |  |  |  |
| V205M | 1.6 | 0.5 to 5.5 | 0.456 |
| D.2) CPT1A model, N=183 |  |  |  |
| PL | 1.9 | 0.4 to 9.6 | 0.429 |
| LL | 2.3 | 0.4 to 13.2 | 0.351 |
| D.3) Integrative model, N=183 |  |  |  |
| V205M | 1.6 | 0.5 to 5.4 | 0.486 |
| PL | 1.9 | 0.4 to 9.7 | 0.425 |
| LL | 2.2 | 0.4 to 12.8 | 0.376 |
| **B) Non-Febrile Seizures**† |  |  |  |
| E.1) V205M model, N=183 |  |  |  |
| V205M | 1.3 | 0.4 to 4.3 | 0.680 |
| E.2) CPT1A model, N=183 |  |  |  |
| PL | 4.5 | 0.5 to 37.3 | 0.160 |
| LL | 7.4 | 0.9 to 64.3 | 0.069 |
| E.3) Integrative model, N=183 |  |  |  |
| V205M | 1.2 | 0.4 to 4.1 | 0.778 |
| PL | 4.5 | 0.6 to 37.4 | 0.160 |
| LL | 7.3 | 0.8 to 63.5 | 0.071 |
| PP – Homozygous wildtype for CPT1A p.P479L, baseline CPT1A measurement in regression model  PL – Heterozygous for CPT1A p.P479L  LL – Homozygous for CPT1A p.P479L  † three participants removed that reported seizures as “possible.” | | | |
