# Supplementary material for: A mild phenotype associated with KCNQ1 p.V205M mediated long QT syndrome in First Nations children of Northern British Columbia: effect of additional variants and considerations for management
Source: Front Pediatr. 2024 May 31;12:1394105. doi: 10.3389/fped.2024.1394105 (PMC11176454; doi:10.3389/fped.2024.1394105)
Supplement: Supplementary file 3 [file Table3.docx]

**Supplementary Table 3.** Logistic regression analysis of LOC events by mQTc categories among p.V205M positive individuals.

| **Model** | **OR** | **95% CI** | **p Value** |
| --- | --- | --- | --- |
| **LOC by mQTc**†, N=39 | | |  |
| 441-460ms | 1.13 | 0.2 to 6.0 | 0.891 |
| >460ms | 1.25 | 0.3 to 6.2 | 0.785 |
| Baseline <440ms, constant = 0.44  † one participant removed that reported LOC event (seizure) as “possible.” | | | |
