# Supplementary figures and images for: A mild phenotype associated with KCNQ1 p.V205M mediated long QT syndrome in First Nations children of Northern British Columbia: effect of additional variants and considerations for management
Source: Front Pediatr. 2024 May 31;12:1394105. doi: 10.3389/fped.2024.1394105 (PMC11176454; doi:10.3389/fped.2024.1394105)

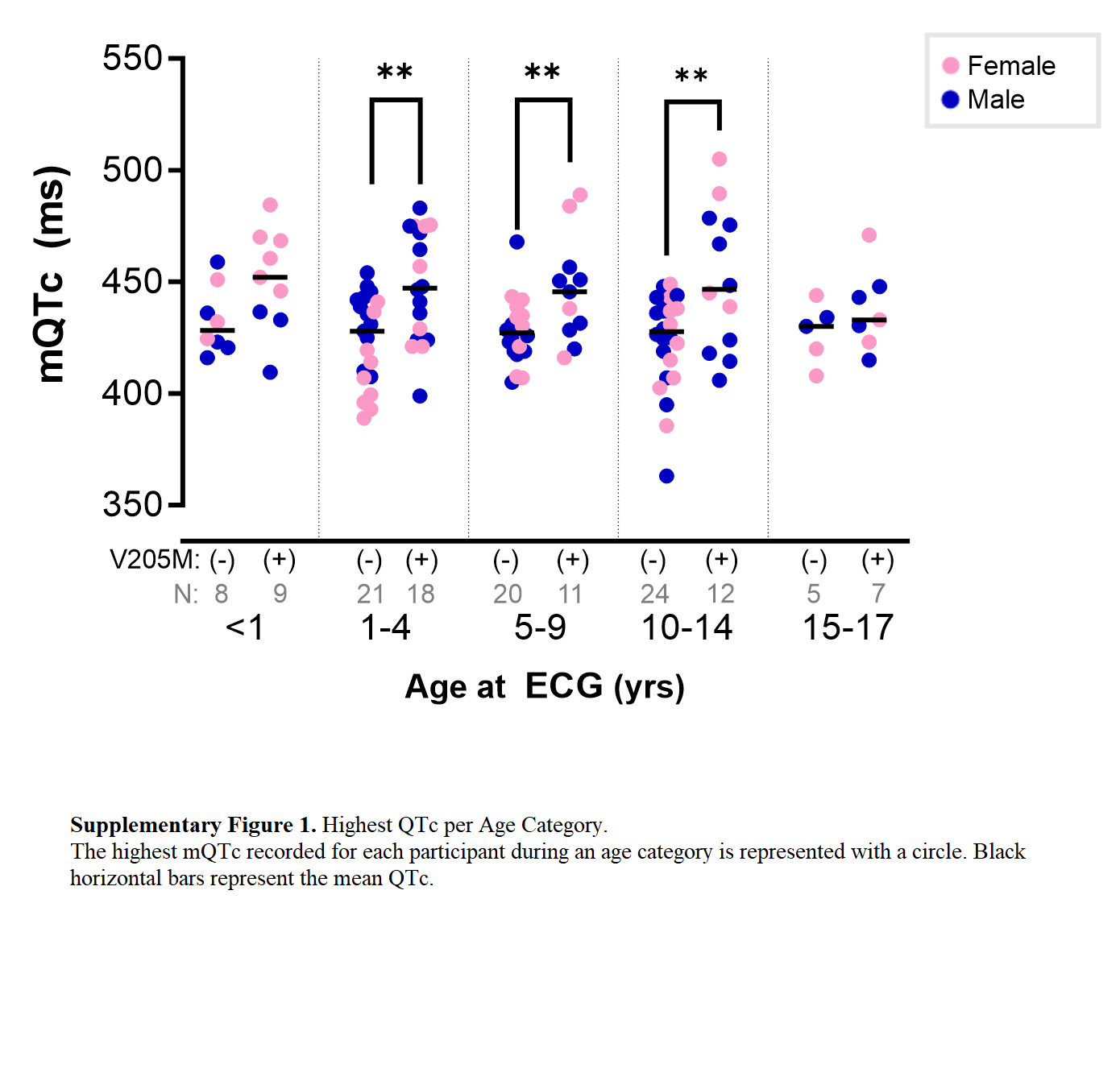

Supplement: Supplementary file 4 [file Image1.tif]

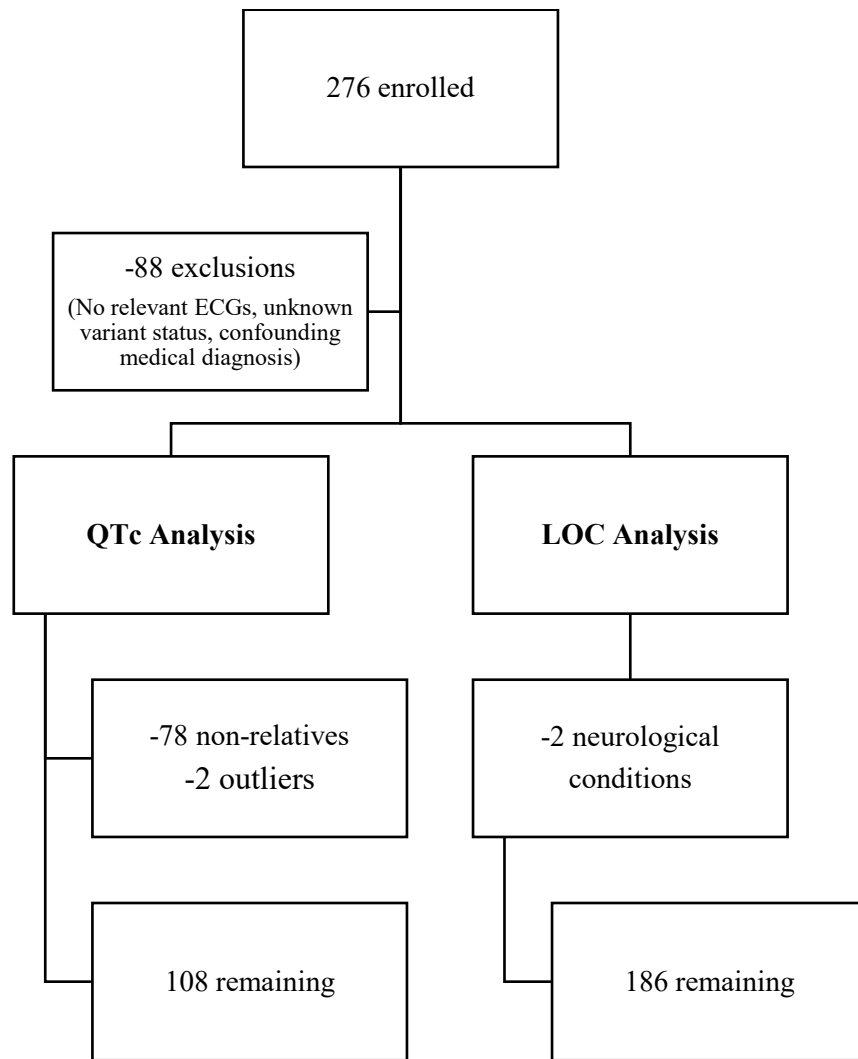

**Supplementary Figure 2.** Participant enrollment and exclusions.

Supplement: Supplementary file 5 [file Image2.pdf]
